# Thermal and Mechanical Characterization of EMA-TEGDMA Mixtures for Cosmetic Applications

**DOI:** 10.3390/polym10030256

**Published:** 2018-03-01

**Authors:** Ruben Donoso, Jose Antonio Reina, Marta Giamberini, Silvia De La Flor, Francesc Ferrando, Pierfrancesco Cerruti

**Affiliations:** 1 Department of Analytical and Organic Chemistry (DQAQO), Universitat Rovira i Virgili, C/Marcel.lí Domingo, 1, 43007 Tarragona, Spain; ruben.donoso@estudiants.urv.cat; 2 Department of Chemical Engineering (DEQ), Universitat Rovira i Virgili, Av. Països Catalans, 26, 43007 Tarragona, Spain; 3 Department of Mechanical Engineering (DEM), University Rovira i Virgili, Av. Països Catalans, 26, 43007 Tarragona, Spain; silvia.delaflor@urv.cat (S.D.L.F.); f.ferrando@urv.cat (F.F.); 4 Institute for Polymers, Composites and Biomaterials (IPCB-CNR), Via Campi Flegrei, 34, 80078 Pozzuoli (NA), Italy; pierfrancesco.cerruti@ipcb.cnr.it

**Keywords:** methacrylate monomers, cosmetics, polymerization rate, thermal properties characterization, mechanical properties characterization

## Abstract

Mixtures of methacrylic polymers are the most common materials for making composites to be used as resins for dental and cosmetic applications. Some of these mixtures are composed by poly(ethyl methacrylate) (PEMA) and poly(methyl methacrylate) (PMMA), which constitute a solid component to be mixed with a liquid component made out of methacrylate monomers. The reaction between the thermal initiator benzoyl peroxyde (BPO) present in the solid component and the activator of the polymerization process, *N*,*N*-dimethyl-*p*-toluidine (DMT) present in the liquid component, gives rise to thermoset materials. In the present study, different liquid formulations composed by a mixture of two methacrylic monomers, ethyl methacrylate (EMA) and triethylene glycol dimethacrylate (TEGDMA) for cosmetic applications, were prepared and characterized, using a commercial powder (POW) composed by PEMA and PMMA. With the aim of improving workability during final application of the material, it was necessary to slow down the polymerization rate of liquid formulations. Their thermal behavior was investigated by differential scanning calorimetric (DSC) in order to check the polymerization rate. Thermal stability of final materials was determined by thermogravimetric analysis (TGA). Dynamic mechanical thermal analysis (DMTA), microindentation hardness and impact tests were performed on final materials, to assess their performance with respect to standard formulation. The combination of thermal and mechanical properties allows choosing which formulations could be suitable for use in cosmetics.

## 1. Introduction

Acrylic and methacrylic monomers are known to be among the most reactive monomers which polymerize by a free-radical mechanism. The availability of a large group of monomeric materials offers the possibility of preparing materials with a wide range of physical properties adaptable to the requirements of many different applications. This feature, together with the remarkable chemical, optical and mechanical properties of the polymers obtained, explains the great commercial success for the resins of acrylic and methacrylic monomers based on thermal or photochemical curing processes [1,2].

Polymers based on acrylic and methacrylic esters are widely used in the formulation of varnishes, paints, adhesives, coatings or composites [3]. Colored products of great beauty can be produced by the incorporation of dyes and pigments into the mixture [4]. Among the properties that make acrylic and methacrylic polymers very important are their mechanical strength, optical properties and extremely good resistance to weathering. Methacrylate polymers, mainly represented by PMMA, have been produced industrially since 1930. The total acrylic glass market in Europe is about 53,000 tons per year. Over 50% of this amount is used for additional processes of the creation of acrylic glasses and molded PMMA components. The remaining quantity is distributed as paints, adhesives, additives, etc. [1,5]

Other methacrylic polymers such as PEMA are produced in lower amounts, and have been used mainly in cosmetics and dental resins. In fact, PEMA could replace PMMA in many of these applications. Due to the fact that PMMA cannot be directly molded into a dental formula by thermal treatment, it is mixed with PEMA as a powder component and a liquid mixture composed by methacrylate monomers to make a processable material. During polymerization process of the mixture of liquid and powder components, the molded material is cured to get the final product [6,7,8,9].

In the case of the cosmetic industry, the use of acrylic resins has been increasing in recent decades. These resins are mainly composed of polymers, oligomers, monomers, inorganic or organic pigments and also polymerization initiators (thermal or photochemical) [10]. In some cases, these resins are formed by two components (one liquid and the other solid in powder form), which are mixed at the time of application, getting a thermoset material. Originally, methyl methacrylate (MMA) was used; nevertheless, it is a strong sensitizer and can cause allergic dermatitis [11,12,13]. This fact led to the use of a mixture of reactive PMMA and PEMA as a powder component, instead of MMA [14].

**Figure 1:**
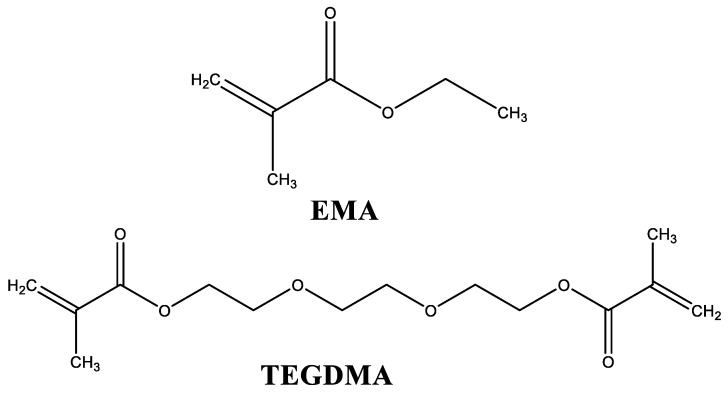
Structures of the main components of liquid formulations. EMA: ethyl methacrylate; TEGDMA: triethylene glycol dimethacrylate.

In the present study, different liquid formulations for cosmetic applications were prepared and characterized. They were cured using the same powder component, constituted by PEMA, PMMA, and BPO, which acts as a thermal initiator [15,16]. The studied liquid formulations are mainly composed by a monofunctional monomer (EMA) and a difunctional monomer (TEGDMA). Their structures are shown in Figure 1.

**Figure 2:**
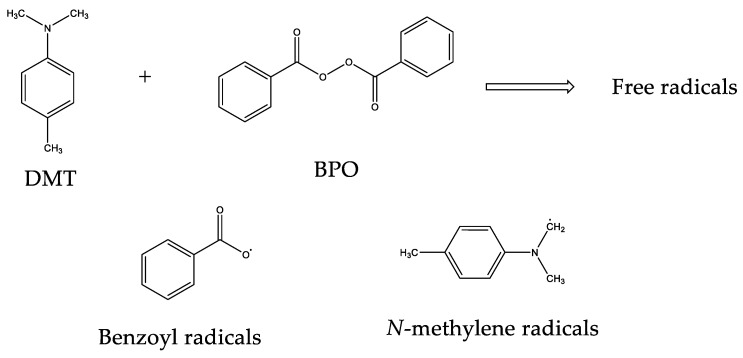
Free radicals generated during the initiation of polymerization process. DMT: *N*,*N*-dimethyl-*p*-toluidine; BPO: benzoyl peroxide.

In addition, liquid formulations also contain an activator of the polymerization process, which is DMT. By mixing these two components at room temperature, the activator reacts with the thermal initiator, generating free radicals (Figure 2) [16,17].

**Figure 3:**
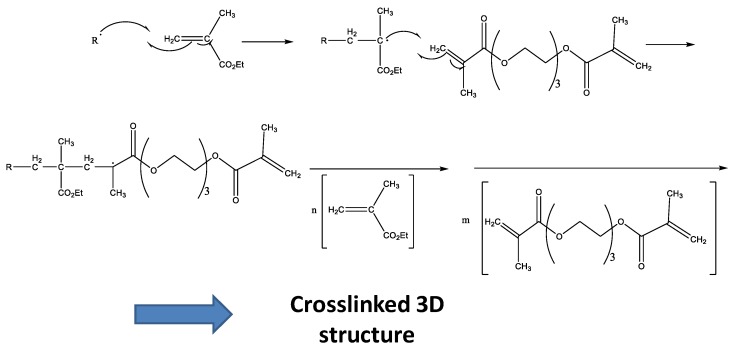
Scheme of the random polymerization process.

These radicals react with the double bonds of methacrylate groups of each monomer present in liquid formulations, giving rise to a random polymerization with more EMA and TEGDMA molecules, and finally lead to a crosslinked network (Figure 3). This final material may present different crosslinking degrees according to the monomer composition in the liquid formulation [17,18].

The objective of this study is to improve the workability during the curing process of the final material for cosmetic applications; that is, slowing down the curing process without significantly altering the performances of the final material. As a matter of fact, the commercial product needs to be manually shaped during its application while curing; therefore, the time required for application should be conveniently adjusted. For this reason, first we determined the effect of different amounts of EMA, TEGDMA and DMT on the polymerization rate of experimental liquid formulations. A commercial liquid formulation was taken as the reference system, which was first reproduced. Polymerization rate of liquid formulations was investigated by thermal analysis and we found that it can be properly varied by changing the EMA/TEGDMA ratio. Afterwards, we analyzed the resulting materials by dynamic mechanical thermal analysis (DMTA), microindentation hardness and impact tests. Results showed that the mechanical properties of materials obtained from slowed-down formulations did not change significantly. The combination of thermal curing rate and resulting mechanical properties was proposed as a criterion to choose which liquid formulations could be suitable for use in cosmetics.

## 2. Experimental Section

### 2.1. Materials

The materials used in this research were prepared from the compounds listed as follows: EMA was supplied by Merck (Darmstad, Germany), TEGDMA was supplied by Esschem Europe (Seaham, UK), DMT and 2-Hydroxy-4-methoxybenzophenone (B-3, which acts as a UV filter, to prevent yellowing of the final material) were supplied by Sigma-Aldrich (Darmstad, Germany), BPO was supplied by Fluka (Darmstad, Germany) and porcelain powder (POW), which contains a mixture of polymethyl methacrylate (in a range of 5–15%), polyethyl methacrylate (in a range of 85–95%), inorganic pigments and BPO (in a range of 0.5–1%), was supplied by the company Laboratorios Bel Cosmetic S.L. (Barcelona, Spain). All of them were used as received, without further purification. Liquid formulations were magnetically blended at room temperature for two hours, and were stored without any exposure to light. Table 1 shows the different liquid formulations prepared and their compositions in mass %.

### 2.2. Experimental Procedures

#### 2.2.1. Procedure for Checking the Curing Process for Liquid Formulations

- In a vial, about 200 mg of a liquid formulation + about 2 mg of BPO were weighted and quickly dissolved at room temperature;
- When the solution was complete, the curing process started, so this vial needed to be kept in an ice/salt bath at –15 °C in order to stop this polymerization process;
- While the DSC device was equilibrated at –50 °C, a sample of the solution was prepared in the crucible in order to introduce it quickly into the DSC oven;
- Finally, after sample introduction into the DSC oven, it was equilibrated at –50 °C a few minutes more and then a dynamic experiment was performed up to 200 °C, in order to check the curing process of the liquid mixture.

#### 2.2.2. Procedure for Preparing Samples of Final Materials for Mechanical Testing

- In a vial, around 10 mL of each liquid formulation was dropped with around 6 g of POW;
- This mixture was stirred quickly at room temperature with a glass bar;
- When the solution achieved the dough state, the mixture was poured over the silicone mold in order to fill it completely;
- The mixture was kept at room temperature overnight in the silicone mold in order to complete the curing process of the materials.

### 2.3. Measurement Techniques

The thermal curing process of each formulation was studied by differential scanning calorimetric (DSC) technique. Both dynamic and isothermal DSC experiments were run under nitrogen flow (100 mL/min) with a Mettler Toledo (Greifensee, Switzerland) DSC-822e instrument. For dynamic experiments, the temperature range was between –50 and 200 °C, at a heating rate of 10 °C/min. For isothermal experiments, the temperature was 25 °C during a total time of 90 min. Each experiment was analyzed three times with standard crucibles in a mass range between 5 and 10 mg.

Thermal stability of the cured materials was checked with a Mettler Toledo (Greifensee, Switzerland) TGA/SDTA851e/LF/1100 device, in the temperature range between 30 and 600 °C, at a heating rate of 10 °C/min using around 10 mg of sample in a nitrogen atmosphere (100 mL/min). The percentage assumed as starting of material degradation was 5% of mass loss.

Dynamic mechanical thermal analysis (DMTA) were carried out with a TA instruments (New Castle, DE, USA) DMA Q800 analyzer. Samples were prepared in a silicon mold as explained above to obtain prismatic rectangular samples of about 35 mm (length) × 7.3 mm (width) × 1.5 mm (thickness). Three point bending clamp (15 mm span length) was used to operate dynamically at 3 °C/min from 30 to 160°C at a frequency of 1 Hz and an oscillation amplitude of 10 μm. *T*g was determined as the maximum of tan δ.

Young’s modulus (*E*) was determined under flexural conditions at 35 °C, with the same clamp and geometry samples, applying a force ramp at constant load rate of 3 N/min, from 0.001 to 3 N. *E* was calculated using the slope of the load deflection curve in accordance with the following Equation (1):

(1) $$E=\frac{L^{3}\cdot m}{4\cdot b\cdot t^{3}}$$

where *E* is the elastic modulus of the sample (MPa), *L* is the support span (mm), *b* and *t* are the width and the thickness of test sample (mm) and *m* is the gradient of the slope for the load deflection curve (∆*F*/∆*L*, N/mm). Three samples of each material were analyzed and the results were averaged.

Microindentation hardness was measured with a Wilson Wolpert (Aachen, Germany) 401MAV device with a micro-Vickers indenter following the ASTM E384-16 standard procedure. 25 or 10 g load was applied depending on the tested sample. In order to obtain the microindentation hardness, 15–20 micro-Vickers indentations were performed on each specimen (three specimens per formulation were analyzed). A statistical calculation with a 95% confidence level was then carried out to determine the upper and lower limits. The Vickers hardness number (*HV*) was calculated from the following Equation (2):

(2) $$HV=\frac{1.8544\cdot F}{d^{2}}$$

where *F* is the load applied to the indenter in kgf and *d* is the arithmetic mean of the length of the two diagonals of the surface area of the indentation measured after load removal in mm.

Aging tests were carried out into a CLIMACELL (Munich, Germany) chamber with temperature and humidity control. A set of mercury-vapor fluorescent tubes was also coupled to the chamber, in order to emit UV-Visible irradiation (around 365 nm); using rectangular samples about 25 mm (lenght) × 12 mm (width) × 2.3 mm (thickness), cured in the same way as described in Section 2.2.2. The distance between the lamp and the samples was adjusted to 10 cm. The selected aging conditions were 25 °C as a constant temperature, 90% of relative humidity and the lamp was switched on for a total time of one week. According to ASTM D 4508-05, impact tests on pre-aged and post-aged samples were performed at room temperature by means of a Zwick 5110 impact tester (Kennesaw, GA, USA). The impact strength (*IS*) was calculated from the energy absorbed by the sample upon fracture as Equation (3):

(3) $$IS=\frac{E-E_{0}}{S}$$

where *E* and *E*_0_ are the energy loss of the pendulum with and without sample respectively, and *S* is the cross-section of the samples. For each material, 8 determinations for pre-aged and post-aged samples were made and the results were averaged with a confidence level of 95%.

## 3. Results and Discussion

### 3.1. Dynamic DSC Study of the Thermal Curing Process for Liquid Formulations:

In order to understand and improve the behavior of this kind of system to be used for cosmetic applications, we chose a commercial formulation as a reference system. Therefore, first we tried to reproduce the composition of the commercial formulation. In this way, we could establish the reference composition to be modified and prepare fresh samples for our analyses, the behavior of which was not altered by storage conditions and time. Afterwards, we decided to improve the curing behavior by properly varying the proportion of the two employed monomers (EMA and TEGDMA) and the activator of the polymerization process (DMT).

**Figure 4:**
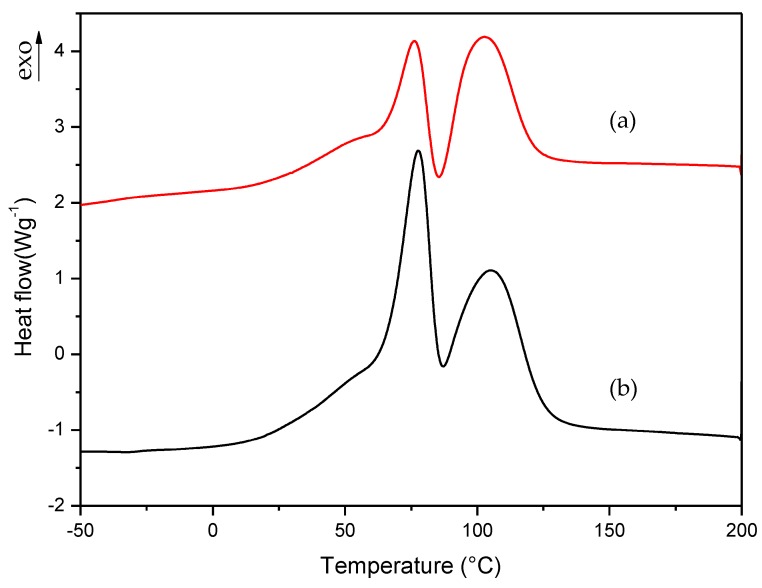
Dynamic DSC curves for curing process of (a) commercial and (b) A formulations.

To check the thermal behavior of the two methacrylate monomers present in the commercial formulation, we performed the procedure described in Section 2.2.1 for commercial liquid formulation. The same procedure was applied to formulation A; that is, to the formulation that was reproduced with the same composition of commercial formulation. Figure 4 shows the dynamic DSC curves for curing process of commercial and A formulations. In these curves, two exothermic peaks can be clearly observed, which correspond to the curing process of the two monomers present in the formulation (EMA + TEGDMA). The endothermic peak observed between 75 and 100 °C correspond to the partial evaporation of the monomer EMA.

**Figure 5:**
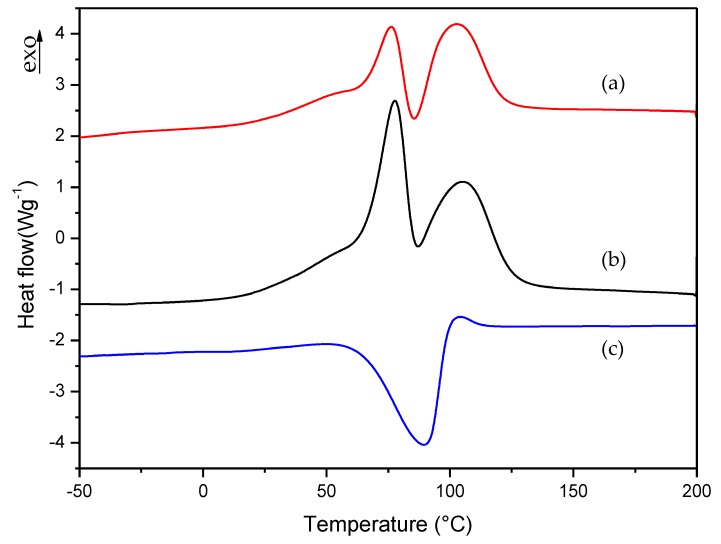
Dynamic DSC curves for curing process of (a) commercial, (b) A and (c) EMA formulations.

To slow down the curing process of liquid formulations, it was necessary to know which monomer had the slowest polymerization rate in order to select how the monomers’ amount could be properly varied to improve the workability of commercial formulation. For this reason, we prepared two additional formulations which contained each monomer separately; i.e., EMA formulation: 98.5% EMA, 1% DMT, 0.5% B-3; TEGDMA formulation: 98.5% TEGDMA, 1% DMT, 0.5% B-3. The curing process of these new formulations was studied with the same procedure described in Section 2.2.1. In this way, we could determine which monomer corresponds to each of the exothermic peaks observed in Figure 4 curves. In the case of TEGDMA formulation the rate of polymerization was so high that it was impossible to observe any peak in the dynamic DSC curve. Figure 5 shows the curing process for commercial, A and EMA formulations.

In the dynamic DSC curve for EMA formulation, a small exothermic peak could be observed, which seems to correspond to the curing process of EMA monomer. In this case, EMA evaporation was more evident due to the lower boiling point of this monomer (about 120 °C) with respect to TEGDMA (higher than 200 °C). However, as can be observed in Figure 5b, the mixture of EMA and TEGDMA exhibits two exothermic peaks; this is due to the different amounts of monomers (83.5% and 15%, respectively) as well as to the high reactivity of TEGDMA, which contributes to increase polymerization rate and the viscosity of the reacting mixture. From the comparison of the above curves, one can guess that EMA has the slowest polymerization rate. Therefore, for slowing down the curing process of commercial formulation, B and C formulations were prepared by increasing EMA amount respect to TEGDMA. The DMT amount was also reduced in D, E, F, and G, H, I formulations in order to check if the curing process could be further slowed down on decreasing the amount of activator in the mixture (see Table 1). The change in the polymerization rate of formulations from A to I was checked by isothermal DSC studies.

### 3.2. Isothermal DSC Study of the Thermal Curing Process for Liquid Formulations

For these experiments, the samples preparation was similar to the procedure described in Section 2.2.1, but in this case a slightly different DSC method was used: after equilibrating the DSC at –50 °C, the second step was a dynamic scan from –50 to 25 °C at a heating rate of 10 °C/min, followed by an isothermal step during 60 min (for commercial, A, B, D, E and G formulations), and during 90 min for C, F, H and I formulations; that is, for the formulations with the slowest polymerization rate observed during the develop of experiments. Table 2 shows the polymerization and residual heat generated in each isothermal process. It should be underlined that the polymerization enthalpies reported just represent an estimation, since the reaction occurs very fast and, despite careful preparation conditions, starts during mixing of components and before sample insertion into the DSC cell: actually, the faster the reaction, the more amount of heat could not be determined by DSC. The residual heat was determined from a second dynamic scan from 25 to 200 °C at a heating rate of 10 °C/min after each experiment. In addition, the time corresponding to 95% of the polymerization degree is also displayed for each isothermal process (*t* at α_95_).

**Figure 6:**
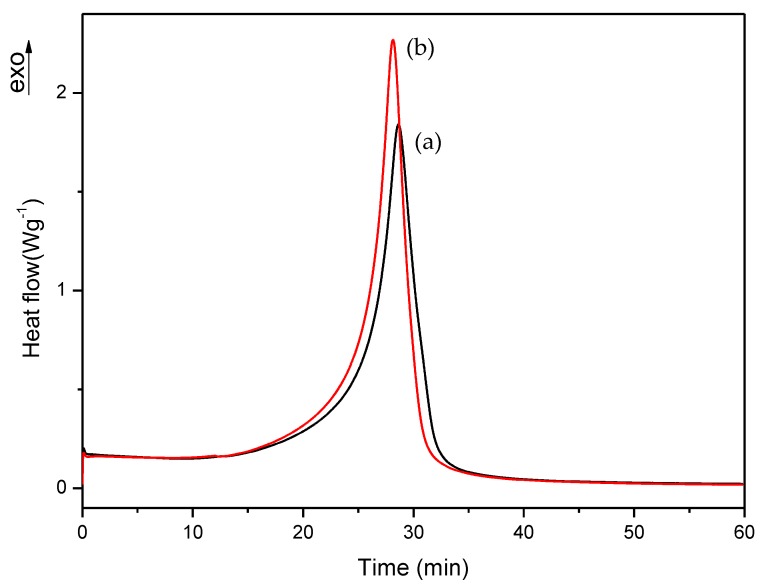
Isothermal DSC curves for (a) commercial and (b) A formulations.

**Figure 7:**
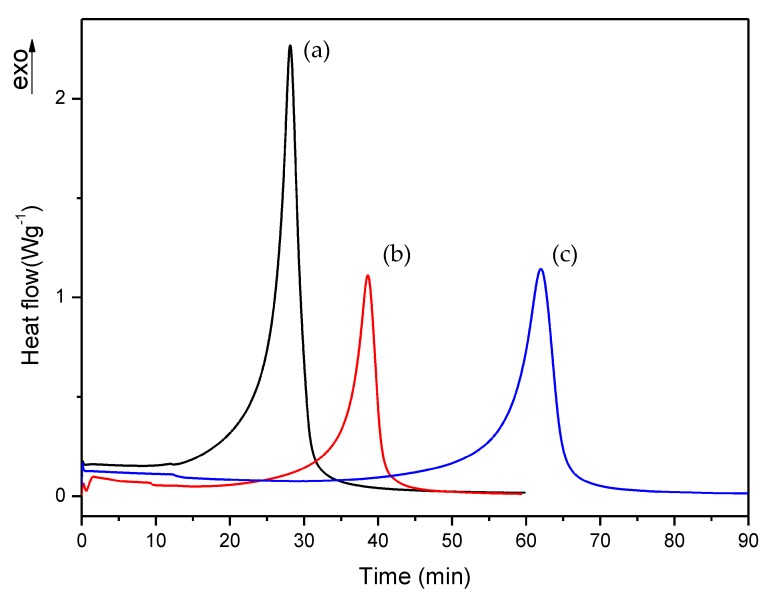
Isothermal DSC curves of (a) A, (b) B and (c) C formulations with 1% DMT.

**Figure 8:**
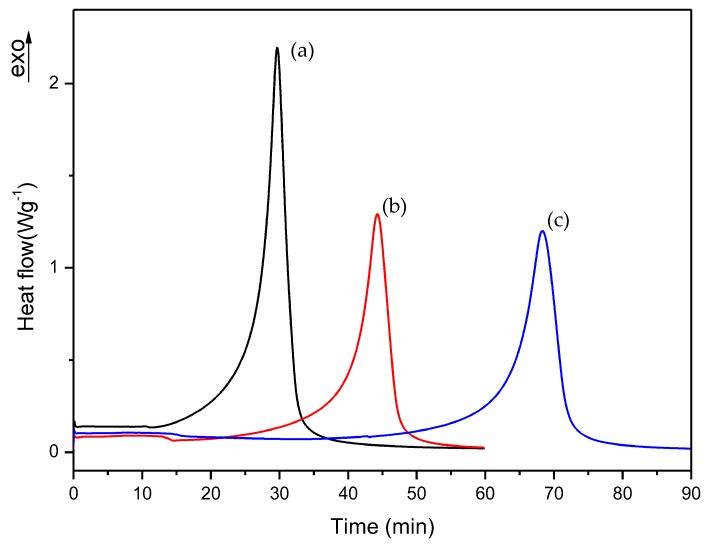
Isothermal DSC curves of (a) D, (b) E and (c) F formulations with 0.75% DMT.

**Figure 9:**
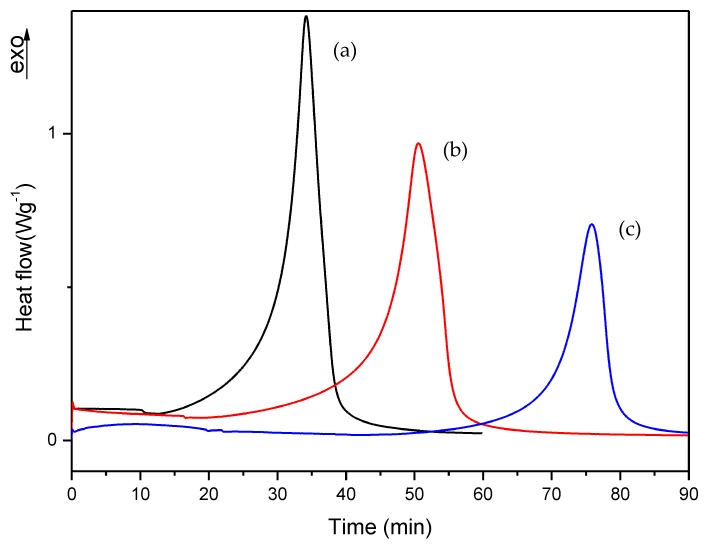
Isothermal DSC curves of (a) G, (b) H and (c) I formulations with 0.50% DMT.

First, we checked the isothermal process of commercial and A formulations, which isothermal DSC curves are shown in Figure 6. Figure 7, Figure 8 and Figure 9 show the isothermal DSC curves comparison of formulations from A to I. Polymerization heat, residual heat and *t* at α_95_ of these samples are displayed in Table 2.

As is evident from Figure 6 and Table 2, the two formulations show similar values, with a residual heat below 10% of the total polymerization heat. Therefore, we can conclude that A formulation exhibited similar behavior to commercial formulations and can be used as a reference in our following studies.

As can be observed, polymerization rate decreases with increasing EMA amount and when DMT amount is reduced. In all studies, we observed that the residual heat was around 10% of the total polymerization heat. According to these results, an increase in polymerization time was observed when the amount of DMT was halved. On the other hand, when EMA amount was increased while DMT amount was kept constant (e.g., A, C or D, F or G, I formulations), a remarkable increase of polymerization time was observed. For instance, on increasing EMA amount by only 10%, polymerization time was practically doubled. These data confirmed that increasing EMA amount in formulations, had a strong influence in slowing down the curing reaction and therefore, could improve the workability for preparing final materials with these liquid formulations.

### 3.3. Thermal and Mechanical Characterization of Materials Prepared with Each Liquid Formulation

In order to check if the slowing-down of curing process of liquid formulations by altering their composition could be detrimental to the features of final materials, their mechanical properties were determined. As an initiator, in this case we used POW, constituted by PEMA, PMMA, and BPO in order to reproduce the working conditions of cosmetic application as faithfully as possible. In this mixture, the proportion of BPO was comparable to the proportion used for performing isothermal DSC experiments of liquid formulations. Therefore, for each final material (cured as needed for final application) it was necessary to determine previously the total time that a mixture of POW and a liquid formulation need for curing completely at room temperature: that is, the time necessary for no appreciable residual heat to be detected. When this condition occurred, it was considered that the material was suitable for mechanical testing. 

First, a small amount of the reference system (formulation A + POW), was cured at room temperature for different times. Afterwards, it was checked by dynamic DSC scans whether residual curing heat could be detected for each curing time. Figure S1 shows the DSC curves related to the evolution at different times of the residual heat for the curing process of A formulation. Table 3 shows the residual heat determined at different times for A formulation. From these results, it was concluded that the material needs more than 5 h to be completely cured. For the rest of the formulations, residual curing heat was evaluated after curing a small amount of each formulation + POW overnight, with the purpose of checking if incomplete curing could negatively affect the mechanical properties of the cured materials. Figure S2 shows the DSC curves related of the residual heat observed from A to F formulations, after overnight curing process. From these results, it is evident that the materials from D to F formulations need more time for curing completely, as expected on the basis of the lower activator amount used; in fact, their determined residual heat (between 10–30 J/g) was around 10% of the total polymerization heat. Therefore, these results suggested that mixtures D–F could exhibit different mechanical properties due to incomplete curing.

For materials prepared from formulations G, H and I (which contained 0.50% DMT), it was observed that, after curing overnight, the samples remained still so soft that it was impossible to use them for checking mechanical properties. For this reason, it was decided to discard these liquid formulations because they were not suitable for practical purposes. 

#### 3.3.1. Thermal Stability Studies of the Cured Materials by TGA

Before checking mechanical properties by DMTA, thermal stability was checked by TGA of A–F cured samples. Figures S3 and S4 show TGA curves of weight loss and weight loss derivatives of each cured material, respectively. Table 4 shows the results obtained from TGA analysis, as the onset temperature of thermal decomposition (corresponding to 5% weight loss) and the maximum temperature of degradation rate for each material.

According to these results, a temperature range of 5% mass loss was approximately in the range 240–280 (°C) for all final materials. Therefore, the maximum temperature selected for DMTA analysis was below 200 °C, in order to ensure the thermal stability of each material during characterization of their mechanical properties.

#### 3.3.2. Thermo-Mechanical Characterization of the Cured Materials by DMTA

The glass transition temperature (*T*g) and Young’s modulus (*E*) were determined for each cured material. Sample preparation was according to the procedure described in Section 2.2.2. Figures S5 and S6 show tan delta and storage modulus comparison for cured materials prepared from liquid formulations with 1% and 0.75% DMT, respectively. Table 5 shows the results of DMTA analyses for each cured material.

As can be seen in Table 5, *T*g values are similar for the different materials; nevertheless, significant differences in Young’s modulus are observed. It seems that, on increasing the amount of EMA, the Young’s modulus progressively decreases; that is, the material turns less stiff. This is not unexpected since the more EMA was added to liquid formulations, the more linear structure should be obtained, with lower crosslinking density.

The amount of DMT activator has also an influence on the value of Young’s modulus (i.e., comparison of A, B, C and D, E, F samples). Indeed, lowering the amount of DMT from 1% to 0.75%, resulted in halving the E value of the final materials. This suggests that, due to lower DMT amount, the structure of the final materials comprises shorter polymer chains, because the BPO initiator does not react completely with DMT activator and therefore generates a lower amount of free radicals. As a result, less rigid materials were obtained. However, modulus reduction is not crucial for the final performance in cosmetic application. On the other hand, *T*g values were slightly affected by both EMA and DMT quantities, due to the higher amount of POW, which has a predominant role in determining the *T*g of the final materials, with respect to the liquid formulations. Figure S7 shows a DSC scan of a POW sample, which shows a *T*g around 80–85 °C.

#### 3.3.3. Microindentation Hardness Characterization of the Cured Materials

**Figure 10:**
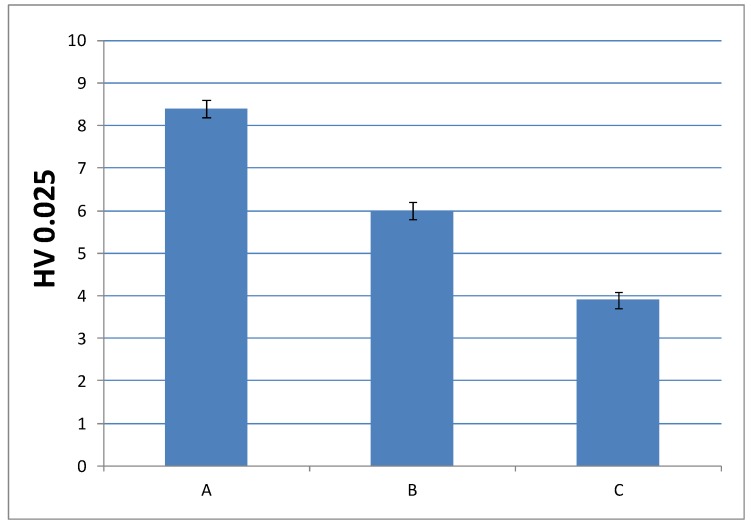
Microindentation hardness (*HV*) of cured materials prepared from liquid formulations with 1% of DMT amount.

**Figure 11:**
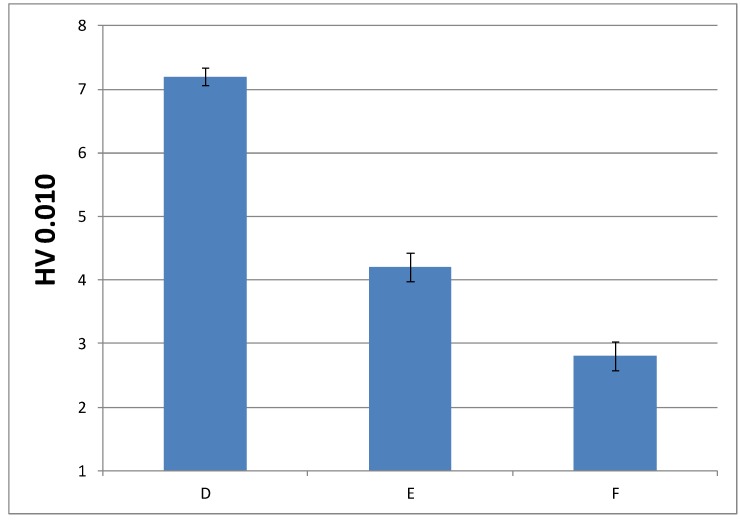
Microindentation hardness (*HV*) of cured materials prepared from liquid formulations with 0.75% of DMT amount.

In order to check whether the change in the composition can be detrimental to the performance of the final material, it was convenient to analyze another mechanical property, such as microindentation hardness; that is, the strength that a material offers against penetration. Figure 10 and Figure 11 show microindentation hardness results observed for each cured material with 1% and 0.75% DMT, respectively.

According to these results, the trend observed in the graph is in agreement with the decreasing values of Young’s modulus; that is, the materials turn less stiff with a lower amount of EMA and DMT due to their internal structure. This leads us to presume that higher amount of EMA and lower amount of DMT in liquid formulations gives rise to a very soft material. Nevertheless, the final microindentation hardness can be still considered acceptable as far as final applications in cosmetic field are concerned.

#### 3.3.4. Impact Strength Characterization of the Cured Material

**Figure 12:**
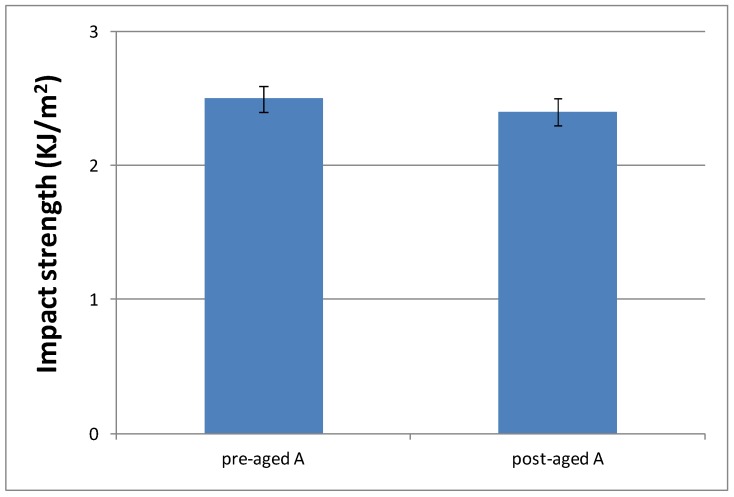
Impact strength values of pre-aged and post-aged materials prepared from A formulation.

Toughness characteristics of cured materials were evaluated by impact test. Previously, 16 samples of cured materials from A formulation were prepared, and eight of them were aged inside the UV chamber. Figure 12 shows impact strength values determined for each material, in order to check if the selected aging conditions had an influence on the material performance.

**Figure 13:**
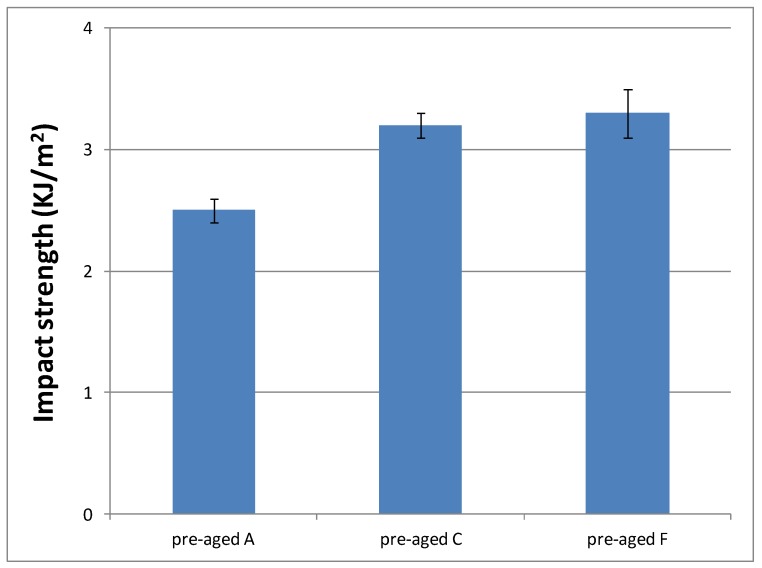
Impact strength values of materials prepared from A, C and F formulations.

As can be seen, aging conditions did not affect the material performance, as evident from the similar values observed. Only surface material changes were observed; that is, aging conditions produced a yellowing phenomenon of the pigments that POW contains (see Figure S8). Therefore, we decided to evaluate only the impact strength of materials cured from the formulations with the slowest curing rate (C and F) without aging, in order to check if they had a different behaviour with respect to the reference system (A formulation). Figure 13 shows these results.

According to these results, similar values of impact strength between formulations with different DMT amount (C and F) were obtained, but they are higher than materials prepared from A formulation. These results are in agreement with their progressively lower crosslinking densities, which make them more tough. That is, the lower the crosslinking density, the higher amount of energy they are capable to dissipate. Therefore, impact strength results make formulations C and F even more suitable for final cosmetic applications. 

## 4. Conclusions

Different formulations for cosmetic applications containing EMA and TEGDMA, as well as DMT as activator of the polymerization process, were investigated. First, by the aid of calorimetric analysis, a commercial mixture was reproduced in order to use it as a reference. Then, we focused our attention on improving the workability of this material by slowing down the curing process without remarkably altering the characteristics of the final product. It was found that, on increasing the EMA amount, the time for workability at room temperature could be usefully improved; on the other hand, the variation of DMT amount did not significantly alter the curing time. As far as the features of the final products are concerned, samples containing the different liquid formulations and commercial POW, constituted by PEMA, PMMA, and BPO, were prepared and cured at room temperature overnight in order to minimize residual curing. These samples were subsequently tested by DMTA and microindentation hardness tester. It was found that *T*g is mainly affected by the presence of a POW component; therefore, it did not exhibit noticeable variations on changing EMA amount; on the other hand, Young’s modulus was remarkably altered on both increasing EMA and decreasing DMT amounts, as expected on the basis of the more linear, shorter chains obtained in these cases. Materials obtained out of both increasing EMA and decreasing DMT amounts exhibit lower microindentation hardness, though still in a suitable range for final application. Experiments on aged samples showed a yellowing phenomenon on the samples’ surface due to the degradation of pigments. Impact strength values confirmed that materials with low curing rate exhibit higher toughness. Therefore, the mechanical properties evaluated are acceptable for cosmetic applications.

## Figures and Tables

**Table 1:** Liquid formulations prepared and their composition in mass %.

Formulation	EMA	TEGDMA	DMT	B-3
A	83.5	15	1	0.50
B	88.5	10	1	0.50
C	93.5	5	1	0.50
D	83.63	15.13	0.75	0.50
E	88.63	10.13	0.75	0.50
F	93.63	5.13	0.75	0.50
G	83.75	15.25	0.50	0.50
H	88.75	10.25	0.50	0.50
I	93.75	5.25	0.50	0.50

**Table 2:** Polymerization heat, residual heat and *t* at α_95_ determined by isothermal DSC studies from commercial to I formulations.

Formulation	Polymerization Δ*H* at 25 °C (10^−2^) (J/g)	Residual Δ*H* (10^−2^) (J/g)	*t* at α_95_ (min)
Commercial	2.4 ± 0.1	0.21 ± 0.01	31 ± 1
A	2.3 ± 0.1	0.23 ± 0.01	32 ± 1
B	2.2 ± 0.2	0.24 ± 0.02	43 ± 2
C	2.3 ± 0.1	0.26 ± 0.05	66 ± 2
D	2.3 ± 0.1	0.27 ± 0.02	34 ± 1
E	2.2 ± 0.1	0.28 ± 0.02	46 ± 1
F	2.0 ± 0.3	0.22 ± 0.02	73 ± 1
G	2.4 ± 0.1	0.30 ± 0.01	37 ± 1
H	2.3 ± 0.1	0.25 ± 0.02	54 ± 1
I	1.7 ± 0.3	0.24 ± 0.01	80 ± 1

**Table 3:** Residual curing heat determined at different times for curing process of A formulation.

Curing time at 25 °C (min)	Residual Δ*H* (J/g)
30	11 ± 1
60	9 ± 1
90	6 ± 2
180	2 ± 1
300	0.5 ± 0.2

**Table 4:** TGA results for each cured material.

Formulation	Onset TGA curve (5% Mass Loss) (°C)	*T*_max_ (°C)
A	273	390
B	251	389
C	256	389
D	267	388
E	236	388
F	245	386

**Table 5:** *T*g and E values for A–F materials as determined by DMTA.

Formulation	*T*g (°C)	*E* (MPa)
A	95 ± 1	900 ± 34
B	95 ± 1	743 ± 37
C	94 ± 1	564 ± 29
D	91 ± 1	436 ± 27
E	91 ± 1	329 ± 22
F	90 ± 1	259 ± 21

## Supplementary Materials

The following are available online at http://www.mdpi.com/2073-4360/10/3/256/s1.

## References

[1] Traxler M., Ackermann J., Juda M., Hirsch D. (2011). Polymethyl Methacrylate (PMMA).

[2] Decker C. (1996). Photoinitiated crosslinking polymerization. Prog. Polym. Sci..

[3] Lazzari M., Scalarone D., Malucelli G., Chiantore O. (2011). Durability of acrylic films from commercial aqueous dispersion: Glass transition temperature and tensile behavior as indexes of photooxidative degradation. Prog. Org. Coat..

[4] Wicks Z.W., Jones F.N., Pappas S.P. (2007). Douglas A. Wicks. Wicks Organic Coatings: Science and Technology.

[5] Bakioglu L. (2003). Polymerization and Characterization of Poly(Ethyl Methacrylate). Master’s Thesis.

[6] He J., Liu F., Vallittu P.K., Lassila L.V.J. (2012). Synthesis of dimethacrylates monomers with low polymerization shrinkage and its application in dental composites materials. J. Polym. Res..

[7] Lee J.K., Choi J.Y., Lim B.S., Lee Y.K., Sakaguchi R.L. (2004). Change of properties during storage of a udma/tegdma dental resin. J. Biomed. Mater. Res. B Appl. Biomater..

[8] Asmussen E., Peutzfeldt A. (1998). Influence of uedma, bisgma and tegdma on selected mechanical properties of experimental resin composites. Dent. Mater..

[9] Muhtaroǧullari I.Y., Doǧan A., Muhtaroǧullari M., Usanmaz A. (2003). Thermal and mechanical properties of microwave and heat-cured poly(methyl methacrylate) used as dental base material. J. Appl. Polym. Sci..

[10] Baki G., Alexander K.S. (2015). Introduction to Cosmetic and Formulation Technology.

[11] Moossavi M., Scher R.K. (2001). Nail care products. Clin. Dermatol..

[12] Marks J.G., Bishop M.E., Willis W.F. (1979). Allergic contact dermatitis to sculptured nails. Arch. Dermatol..

[13] Kanerva L., Henriks-Eckerman M.L., Jolanki R., Estalander T. (1997). Plastics/acrylics: Material safety data sheet need to be improved. Clin. Dermatol..

[14] Ramírez F., Félix M., Romero A., Guerrero A. (2015). Mechanical properties of acrylic nails in the guitar playing. Afinidad LXXII.

[15] Sideridou I.D., Achilias D.S., Karava O. (2004). Reactivity of benzoyl peroxide/amine system as an initiator for the free radical polymerization of dental and orthopaedic dimethacrylate monomers:  Effect of the amine and monomer chemical structure. Macromolecules.

[16] Zoller A., Gigmesa D., Guillaneuf Y. (2015). Simulation of radical polymerization of methyl methacrylate at room temperature using a tertiary amine/BPO initiating system. Polym. Chem..

[17] Vazquez B., Levenfeld B., Roman J.S. (1998). Role of amine activators on the curing parameters, properties and toxicity of acrylic bone cements. Polym. Int..

[18] Vazquez B., San Roman J., Deb S., Bonfield W. (1998). Application of long chain amine activator in conventional acrylic bone cement. J. Biomed. Mater. Res..

