# Supplementary material for: Thermal and Mechanical Characterization of EMA-TEGDMA Mixtures for Cosmetic Applications
Source: Polymers (Basel). 2018 Mar 1;10(3):256. doi: 10.3390/polym10030256 (PMC6415040; doi:10.3390/polym10030256)
Supplement: Supplementary file 1 [file polymers-10-00256-s001.pdf]

polymers

Supplementary material

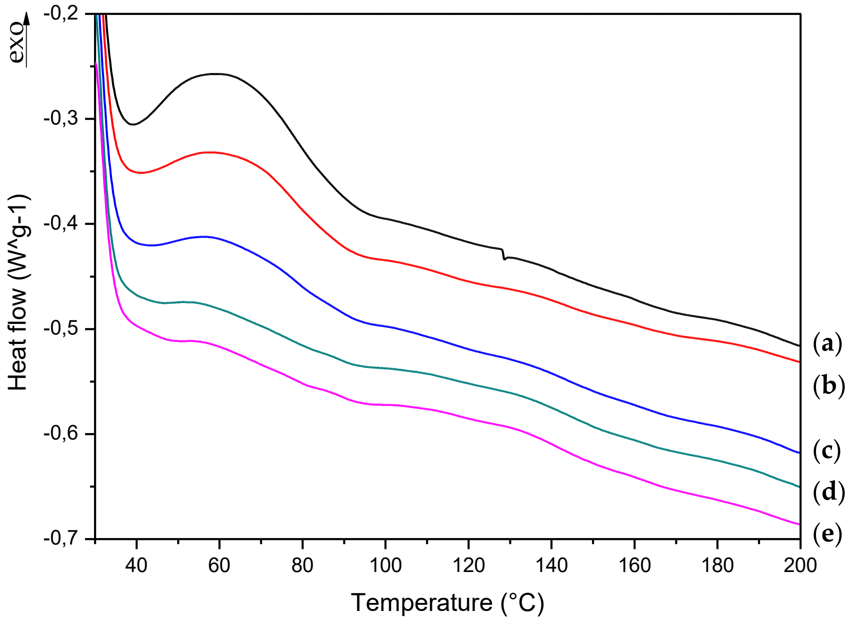

Figure S1. Dynamic differential scanning calorimetric (DSC) curves related to the evolution of the residual heat for A formulation after different curing times at 25°C: (a) 30 minutes, (b) 60 minutes, (c) 90 minutes, (d) 180 minutes and (e) 300 minutes.

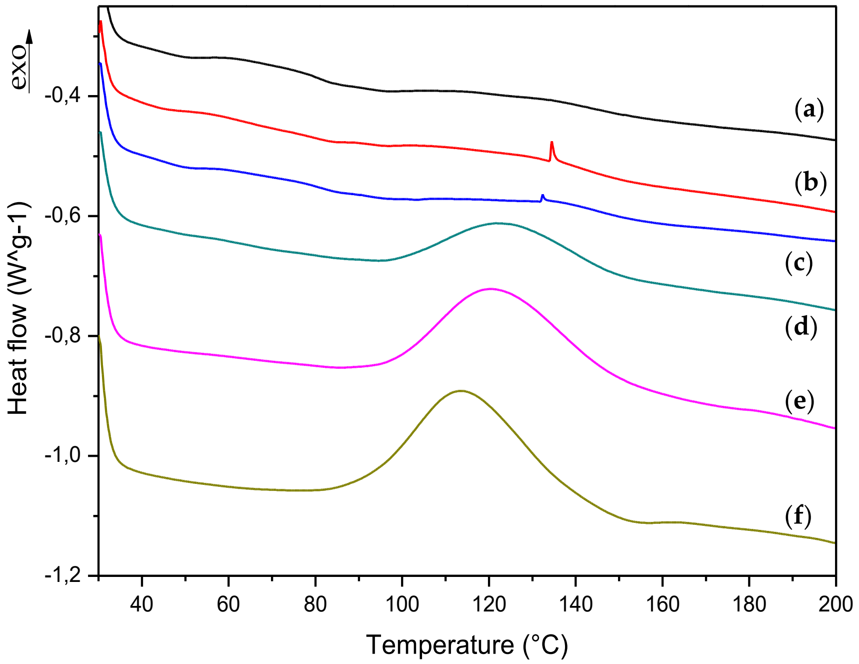

Figure S2. Dynamic DSC curves related to the residual heat observed after curing overnight a sample from (a) A to (f) F formulations.

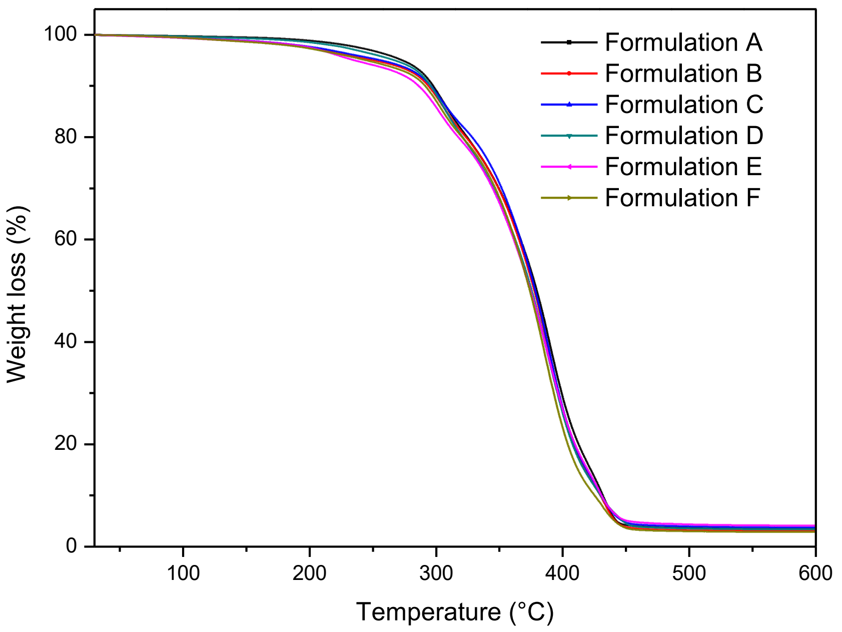

Figure S3. Dynamic thermogravimetric analysis (TGA) curves related to weight loss for final materials prepared from A to F formulations.

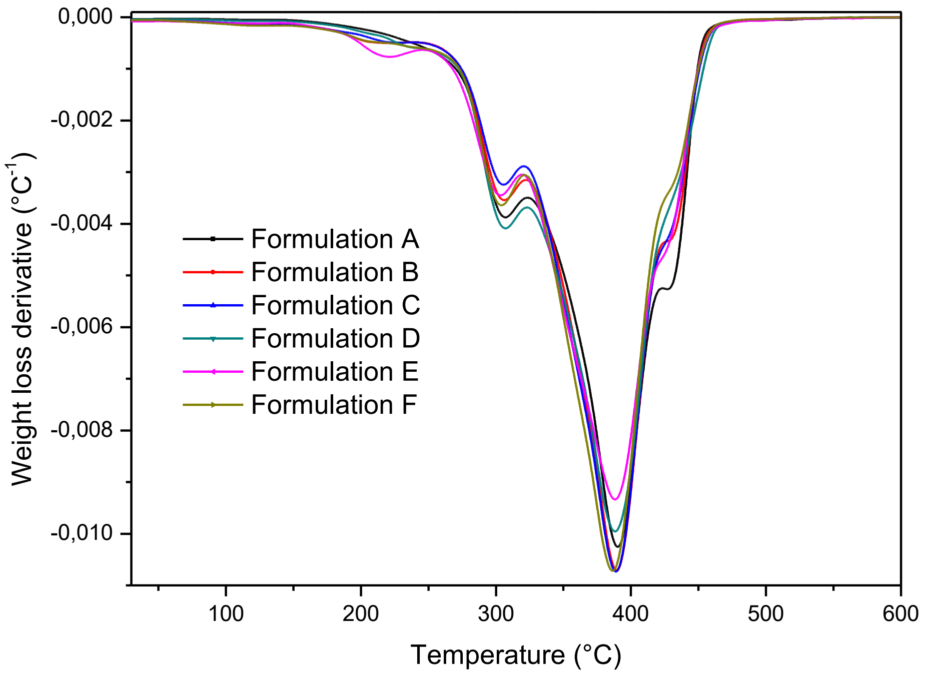

Figure S4. Dynamic TGA curves related to weight loss derivative for final materials prepared from A to F formulations.

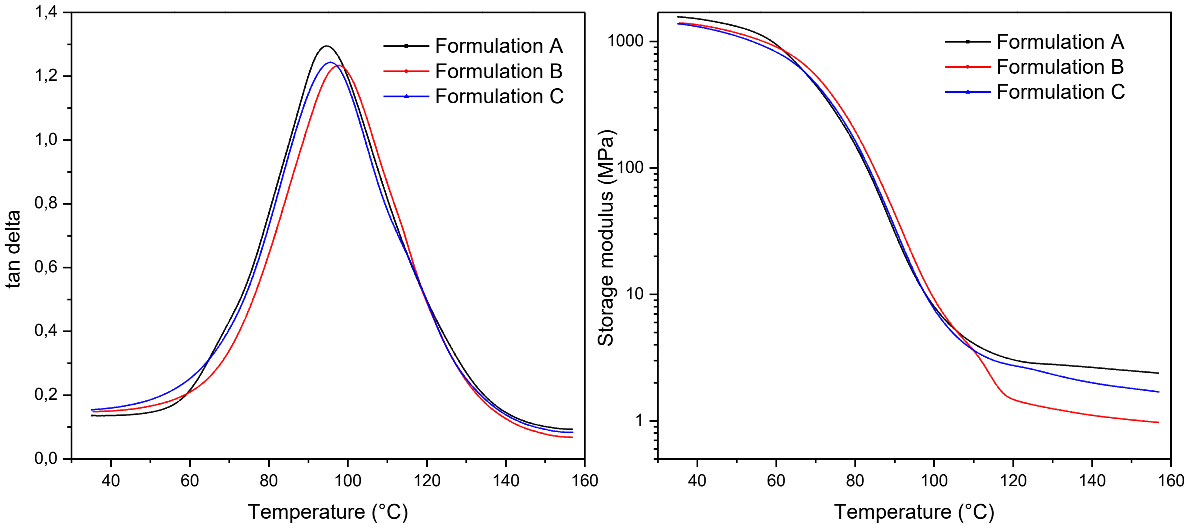

Figure S5. Tan delta and storage modulus comparison for each cured material prepared from A, B and C formulations with 1% *N*,*N*-dimethyl-*p*-toluidine (DMT).

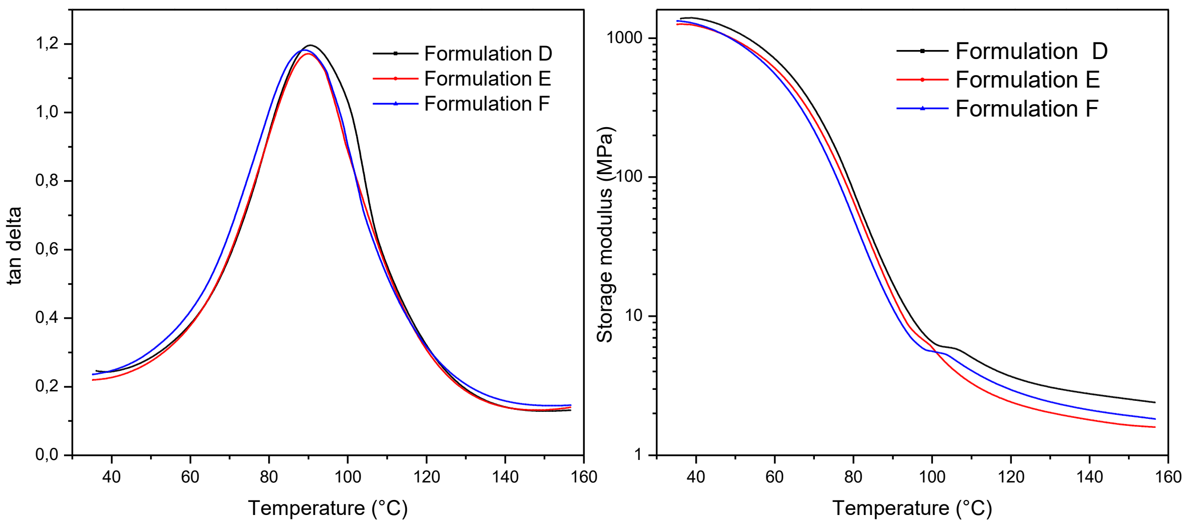

Figure S6. Tan delta and storage modulus comparison for each cured material prepared from D, E, and F formulations with 0.75% DMT.

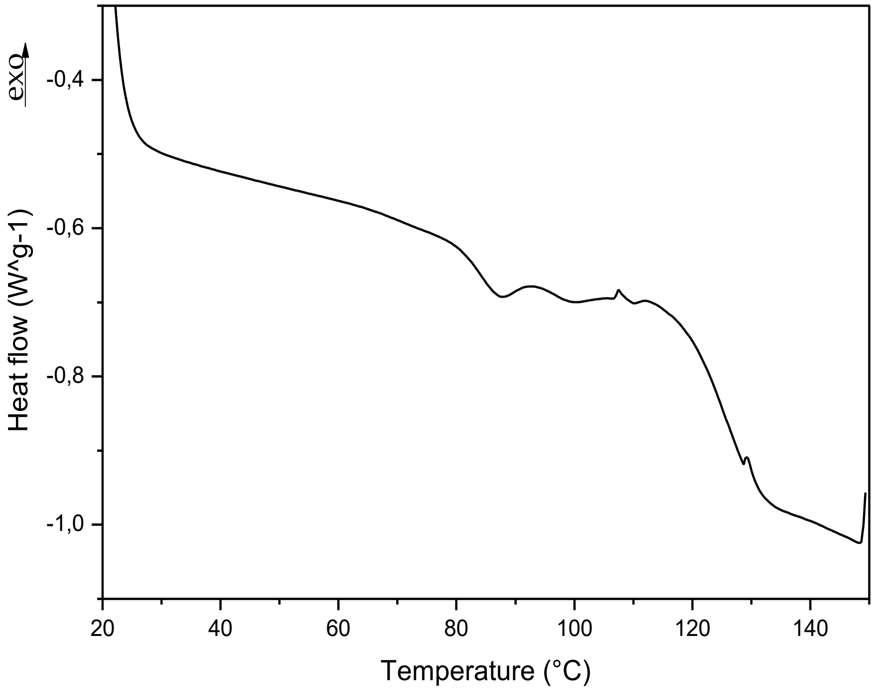

Figure S7. Dynamic DSC curve of *T*g determination for commercial powder (POW).

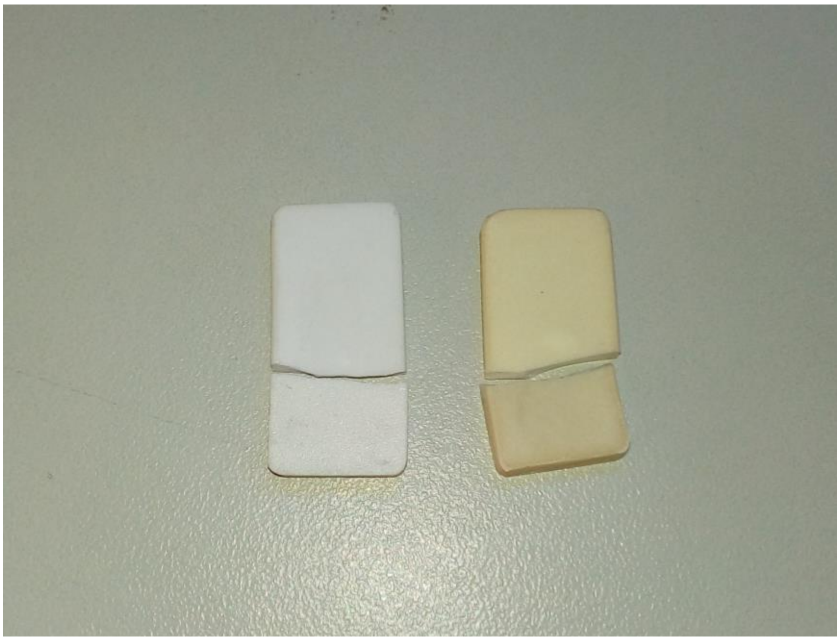

Figure S8. Pre-and post-aged samples of cured materials for impact tests.
